# The influence of middle-aged and older adults’ social capital and education on physical function: evidence from the China Health and Retirement Longitudinal Study

**DOI:** 10.3389/fpubh.2024.1511611

**Published:** 2024-12-04

**Authors:** Tingfai Man, Yongze Zhao, Huaxin Mai, Ying Bian

**Affiliations:** ^1^Department of Public Health and Medicinal Administration, Faculty of Health Sciences, University of Macau, Taipa, Macao SAR, China; ^2^Institute of Chinese Medical Sciences, University of Macau, Taipa, Macao SAR, China; ^3^State Key Laboratory of Quality Research in Chinese Medicine, University of Macau, Taipa, Macao SAR, China; ^4^Unit of Psychiatry, Department of Public Health and Medicinal Administration, Institute of Translational Medicine, Faculty of Health Sciences, University of Macau, Taipa, Macao SAR, China

**Keywords:** middle aged, aged, social capital, education, physical function, CHARLS

## Abstract

**Background:**

Population aging is a major global trend with significant social, economic, and health implications. In China, the increasing aging population presents challenges, including increased chronic diseases and disabilities. Social capital has emerged as vital in determining health outcomes for middle-aged and older adults. This study seeks to examine the impact of social capital and educational attainment on physical functioning in middle-aged and older adults, with particular emphasis on the moderating effect of education within this relationship.

**Methods:**

This study utilized data from the China Health and Retirement Longitudinal Study (CHARLS) from 2018 to 2020, involving 9,497 participants aged 45 and older. Physical function was assessed using the Activities of Daily Living (ADL) and Instrumental Activities of Daily Living (IADL) scales. Social capital was measured in four dimensions: social trust, social support, social participation, and reciprocity. Educational attainment was categorized into four levels: below primary school, primary school, middle school, and high school or above. Cox proportional hazards regression and moderating effect models were used for data analysis, adjusting for demographic and health-related variables.

**Results:**

Our findings highlight the significant roles of social participation (aHR = 0.856, 95%CI: 0.675–0.809) and reciprocity (aHR = 0.700, 95%CI: 0.626–0.784) in improving physical function. Education enhanced the positive effects of social participation (aHR = 0.923, 95%CI: 0.840–0.980). Subgroup analyses showed that social support was a protective factor for females (aHR = 0.857, 95% CI: 0.737–0.998), while social trust negatively affected urban residents (aHR = 1.330, 95%CI: 1.135–1.560).

**Conclusion:**

The findings underscore the importance of social participation, reciprocity, and education in enhancing physical function among middle-aged and older adults. Tailored interventions addressing gender and residential differences are essential to meet the unique needs of various subgroups. Understanding the relationship between social capital, education, and health can inform strategies to improve this population’s well-being.

## 1 Introduction

Population aging is a significant demographic trend observed globally, characterized by an increasing proportion of older individuals within the total population. According to the United Nations’ report on Population Ageing (2019), the number of individuals aged 65 and older is projected to double from 703 million in 2019 to 1.5 billion by 2050, resulting in profound social, economic, and health implications (1). In China, the aging population presents a particularly pressing challenge. By 2021, individuals aged 60 and older accounted for approximately 18.9% of the total population, as reported in the China Statistical Yearbook, and this figure is expected to rise to over 30% by 2050 (2). The implications of population aging are manifold, significantly impacting healthcare systems and pension schemes. As the proportion of older adults increases, there is a corresponding rise in the prevalence of chronic diseases and disabilities, placing considerable strain on healthcare resources.

While a universally accepted definition of social capital remains elusive, various perspectives highlight its significance in social structures and individual behaviors. In the context of healthcare, social capital has emerged as a critical determinant of health outcomes, particularly among middle-aged and older adults. Social capital encompasses the networks, relationships, and norms that facilitate collective action and provide individuals with access to resources and support. Research consistently demonstrates that individuals with higher levels of social capital tend to experience better functional health, which refers to the ability to perform daily activities and maintain independence (3–8). This relationship is especially relevant for older adults, who often encounter challenges related to aging, chronic illnesses, and social isolation (9). Most of the literature about social capital collectively underscores the significant role of social capital in enhancing health outcomes. Cognitive social capital, characterized by trust and mutual support, consistently shows positive effects on both mental and physical health. Structural social capital, involving social networks and participation, also benefits health but can sometimes negatively impact mental health if the social structures are stressful or demanding (10–12). Interventions aimed at boosting social capital have been shown to improve public health outcomes, particularly for individuals with lower socioeconomic status. Overall, the studies highlight that fostering social capital can lead to healthier behaviors and better health, especially among middle-aged and older adults.

The significance of social capital is well-documented; however, its impact on functional health is not uniform across various demographic groups. Educational attainment, a crucial factor in understanding health disparities, may moderate this relationship. Previous research has identified education as a significant determinant of social capital (13). Studies indicate that individuals with higher levels of education tend to exhibit greater trust in others and are more likely to engage actively in social organizations and participate frequently in group activities. Furthermore, education may equip individuals with the knowledge and skills necessary to effectively acquire health information and resources, thereby enhancing their ability to leverage social capital for improved health outcomes. Additionally, higher levels of education are often associated with better socioeconomic status, which can further impact health behaviors and access to healthcare services.

While previous research has established the importance of social capital in influencing health outcomes, there has been limited investigation into how educational levels may affect this relationship. A study examining the influence and health implications of online social capital among middle-aged and older adults in China highlights the growing trend of internet usage for daily social interactions within this demographic (14). The findings indicate that the online social capital accumulated through these interactions can significantly affect health outcomes. Specifically, engaging in online social networks provides middle-aged and older individuals with access to diverse and heterogeneous information, which can contribute to improved health and well-being. It should be noted that this result is affected by the level of education.

As previously noted, the relationship between social capital and health has been extensively studied. However, the interaction between education and social capital remains underexplored, particularly in the context of China. This study aims to address a theoretical gap by investigating the moderating role of educational attainment in the relationship between social capital and functional health among middle-aged and older adults, particularly in the context of an aging population in China. By employing a robust analytical framework, we seek to elucidate how varying levels of education influence the effectiveness of social capital in promoting functional health outcomes. In this study, we hypothesize that educational level can enhance the positive effects of social participation on physical function, especially in individuals who have received higher education. However, the moderating effect of education level on the relationship between social trust and social support and physical function may not be significant. Differences in gender and place of residence may affect the impact of various dimensions of social capital on health outcomes. The level of education may enhance the positive impact of social capital on health outcomes by improving individuals’ ability to access health information and increasing opportunities for social participation. To achieve this, we utilize longitudinal data derived from the China Health and Retirement Longitudinal Study (CHARLS), covering two waves from 2018 to 2020. This article represents the first investigation utilizing the most recent data to examine the moderating effect of educational attainment on social capital among middle-aged and older adults in China. Understanding the interplay between social capital, education, and health is crucial for developing targeted interventions and policies aimed at improving the quality of life for older adults. The findings of this research will provide valuable insights for healthcare practitioners, policymakers, and community organizations striving to enhance the health and well-being of this demographic group.

## 2 Methods

### 2.1 Study population

This study utilized publicly available data collected by the National School of Development at Peking University from the China Health and Retirement Longitudinal Study (CHARLS), an ongoing longitudinal investigation targeting individuals aged 45 and older in China. The CHARLS baseline survey employed a multi-stage probability proportional to size (PPS) random sampling method, incorporating implicit stratification based on indicators such as region, urban/rural characteristics, and GDP *per capita*. In the first stage, all counties and districts in the country, with the exception of Tibet, were ranked according to urban–rural attributes and GDP *per capita* within each of the eight regions. Subsequently, counties or districts were selected with a probability proportional to their population size. In the second stage, three secondary sampling units (village committees or neighborhood committees) were randomly chosen, again with probability proportional to population size, within each sampled county. Following the sampling process outlined above, the baseline sample of CHARLS was distributed across 450 villages and neighborhoods in 28 provinces and 150 districts and counties (15). Data were collected through face-to-face, computer-assisted personal interviews, which is a method that ensures high-quality data collection and participant engagement. CHARLS utilizes international data collection methods to ensure comparability, referencing studies such as the Health and Retirement Study (HRS) in the United States. As a result, CHARLS is representative both nationally and regionally. The national baseline survey was carried out in 2011 (wave 1), followed by subsequent follow-up surveys employing standardized questionnaires in 2013, 2015, 2018, and 2020 (waves 2–5). In each follow-up survey, the original participants were revisited, and new participants were recruited using the same sampling methods to compensate for attrition and maintain representativeness. Comprehensive details regarding the objectives, design, sampling procedures, and questionnaires of the CHARLS are available in other articles (15). The study was conducted following the Declaration of Helsinki, and approved by the Institutional Review Board of Peking University. The IRB approval number for the main household survey is 00001052–11,015, and all study subjects signed an informed consent form. The study followed the Strengthening the Reporting of Observational Studies in Epidemiology (STROBE) guideline for cohort studies (see Supplementary Table S1).

**Figure 1:**
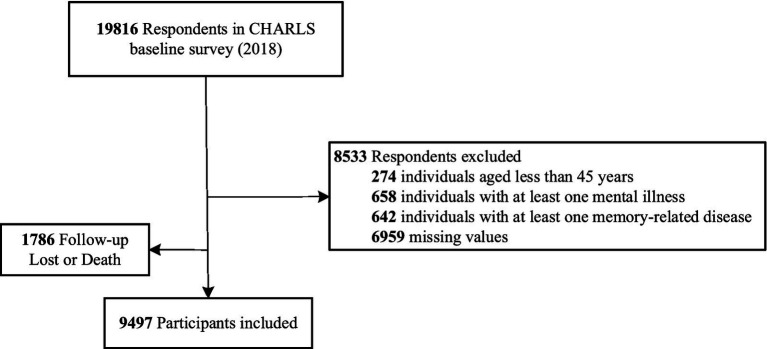
Flowchart of study sample from the china health and retirement longitudinal study.

The follow-up period for the CHARLS data utilized in this study spanned from July 2018 to June 2020. Physical function was assessed as the primary outcome variable, while education level was examined as a moderating variable. The baseline population of CHARLS 2018 was subject to the following exclusion criteria: (1) individuals aged less than 45 years (*n* = 274); (2) individuals with at least one mental illness, including depression, schizophrenia, anxiety disorders, or bipolar disorder (*n* = 658); (3) individuals with at least one memory-related disease, such as Alzheimer’s disease or dementia (*n* = 642). After removing missing values (*n* = 6,959), a total of 11,283 samples were ultimately included. Figure 1 shows a comprehensive overview of the sample processing flow. A total of 9,497 samples from 2018 to 2020 were ultimately included in the construction of the Cox proportional hazards model and the analysis of moderation effects.

### 2.2 Physical function

Physical function was measured by the Activities of Daily Living (ADL) and the Instrumental Activities of Daily Living (IADL) scales. ADLs include dressing, bathing, eating, getting in and out of bed, using the toilet, controlling bowel movements, and taking medication. ADLs include dressing, bathing or showering, eating, getting in and out of bed, using the toilet, and controlling urination and defecation: IADLs include doing household chores, preparing hot meals and shopping for groceries, making phone calls, taking medications and managing money. All questions have 4 options: (1) Do not have any difficulty; (2) Have difficulty but can still do it; (3) Have difficulty and need help; (4) Can not do it. If the answer to any of the ADLs and IADLs is one of the options 2, 3, or 4, it is defined as poor functional physical health; if the answer to any of the ADLs and IADLs is option 1, it is defined as well functional physical health. The scale has been used in research in China and abroad and has shown good reliability and validity.

### 2.3 Social capital and education level

Social capital is measured by four dimensions: social trust, social support, social participation, and reciprocity (16). According to previous studies, China is characterized as a “society of acquaintances,” particularly in rural areas where social capital plays a more significant role in the social economy due to the delayed development of formal institutions (17, 18). If a person’s place of birth is the same as their current place of residence, they may have developed deeper social connections and networks in that area, which can foster a higher level of social trust. Social trust is often linked to the closeness, familiarity, and interdependence within a community. In familiar social environments, individuals are more likely to trust one another due to shared cultural backgrounds, values, and experiences. Therefore, based on this context, place of birth serves as a proxy variable for social trust. Participants whose place of birth matches their current residence are classified as having high social trust, while those whose places differ are classified as having low social trust.

Social support is defined as the exchange of resources between at least two individuals, where at least one party perceives the exchange as aimed at enhancing the recipient’s well-being (19). Social support typically encompasses emotional, material, and informational support that an individual perceives from members of their social network, such as family, friends, and colleagues. Based on this definition, financial support was used to measure social support, setting social support as yes or no based on “whether or not they have received in-kind (buying food, clothes or other stuff regularly for you) or money (Regular payment includes providing living expenses, paying for water, electricity or telephone bill, paying for mortgage/rent or other forms of regular expenses) from their children or whether or not they have pension insurance (3). Due to the challenges of directly measuring emotional support and its inherent subjectivity, it was excluded from this study.

Social participation refers to the involvement in activities that provide interaction with others in a social network, fostering a sense of belonging and community (20–22). Reciprocity, on the other hand, involves the mutual exchange of support and assistance, where individuals not only receive help but also provide it to others, thus creating a supportive network (23). Social participation and reciprocity are judged by the participation in 8 activities in the past month: (1) Attended an educational or training course; (2) Interacted with friends; (3) Played Ma-Jong, played chess, played cards, or went to community club; (4) Went to a sport, social, or other kinds of the club; (5) Took part in a community-related organization; (6) Done voluntary or charity work, of caring for a sick or disabled adult who does not live with you; (7) Caring for a sick or disabled adult who does not live with you; (8) Provided help to family, friends, or neighbors who do not live with you. If at least one of the activities numbered 1 through 5 meets the specified conditions, it is classified as exhibiting Social Participation Behavior; otherwise, it is classified as lacking Social Participation. Similarly, if at least one of items 6, 7, or 8 meets the conditions, it is classified as demonstrating reciprocity; otherwise, it is classified as lacking reciprocity.

Based on the structure of the CHARLS questionnaire, we categorized educational attainment into four levels: below primary school, primary school, middle school, and high school or above. Individuals with less than a primary school education are classified as illiterate; otherwise, they are considered non-literate.

### 2.4 Covariates

Referred to prior research, the covariates in this study were categorized into demographic variables and risk factors. Demographic variables included (1) Age, (2) Gender (Male or Female), (3) Marital Status (Divorced/Separated/Widowed/Never Married or Married/Cohabitated), (4) Residence (Rural or Urban), (5) Economic capacity (Yes or No) if participants have income apart from their family or friends’ support or pension. Risk factors are as follows: Sleep duration is categorized into three groups based on hours: insufficient sleep (less than or equal to 6 h), sufficient sleep (more than 6 h but less than or equal to 8 h), and excessive sleep (more than 8 h). If participants have at least one chronic disease such as Hypertension, Dyslipidaemia and Diabetes, they are classified as having chronic diseases, otherwise, they are classified as having no chronic disease. Smoking and drinking behaviors were self-reported by participants based on their current experience.

### 2.5 Statistical analysis

All statistical analyses were conducted using Stata 17.0 software (StataCorp LLC, College Station, TX, USA). First, descriptive statistics were used to summarize the baseline characteristics of the study participants. Continuous variables were expressed as mean ± standard deviation (SD), and categorical variables were presented as percentages. Continuous variables were expressed as mean ± standard deviation (SD), and categorical variables were presented as frequencies and percentages. Group comparisons (physical function) were performed using *t*-tests and chi-square. Group comparisons (physical function) were performed using *t*-tests and chi-square tests. To investigate the relationship between social capital, educational level, and physical functioning, we conducted a cohort study with 2018 (wave 4) participants as the baseline population. Social capital was categorized into 4 dimensions for risk-proportional Cox regression analysis. The exposure variables included social capital and educational level, while the outcome event was poor physical function. Cox regression, in contrast to logistic regression, was utilized to identify cause-and-effect relationships. To account for the influence of confounders on outcomes, we adjusted the model for demographic information, as well as participants’ smoking, drinking, sleep duration, disability, and chronic disease status.

Considering the potential influence of educational status, we conducted a moderation effect analysis using the Cox regression model described above. Moderation is a significant methodological concept in social science research and serves as a valuable tool for investigating the interplay among multiple variables (24). When the relationship between the dependent variable Y and the independent variable X (specifically, the magnitude and direction of the regression slope) varies in response to changes in a third variable Z, it is posited that Z exerts a moderating effect on the relationship between X and Y. In this study, social capital is designated as the primary effect variable, while education is employed as the moderating variable. The Cox moderated effect model is as follows:


$$h\left(t,x\right)=h_{0}\left(t\right)\exp\left(\begin{array}{l} \beta_{1}\mathrm{Socialcapital}+\beta_{2}\;\mathrm{Education}\\ +\beta_{3}\;\mathrm{Socialcapital}\times \mathrm{Education}+\ldots +\beta_{m}X_{m} \end{array}\right)$$

Where *h(t, X)* is the risk function of an individual with covariate *x* at time *t*. *t* is the survival time, and *X = (X_1_, X_2_,…, X_m_)* is the relevant factor that may influence survival time, commonly referred to as covariates. h0(*t*) is the risk function when all covariates are set to zero, known as the baseline hazard function. *β = (β_1_, β_2_,…, β_m_)* is the regression coefficient of the Cox model, which consists of a set of regression parameters to be estimated.

In conducting a moderated effects analysis, centering (i.e., subtracting the mean from the variable) for independent and moderating variables is necessary to reduce the covariance between the interaction term and these variables. Centering corrects these biases without affecting model estimation. Thus, we centered the main and moderated effect variables to create a centered error correction moderated effects Cox model. Finally, we conducted subgroup analyses based on gender and residence type to examine their influence on the results. Due to the presence of missing values for covariates such as sleep duration, smoking, and alcohol consumption (*n* = 648), we used multiple imputation techniques to address the missing data and assessed the robustness of the results. All statistical tests were two-sided, with a *p*-value of less than 0.05 considered statistically significant.

## 3 Results

### 3.1 Baseline sample characteristics

The baseline characteristics of participants in the 2018 wave of the China Health and Retirement Longitudinal Study (CHARLS) are presented in Table 1. The study sample comprised 9,497 respondents, of whom 5,632 (59.3%) were women and 6,132 (64.6%) resided in rural areas. The mean age of participants with poor physical function was 65.84 years (SD = 9.937), whereas those with good physical function had a mean age of 61.51 years (SD = 9.302), indicating a statistically significant difference (*t* = 481.869, *p* < 0.001). Among participants with poor physical function, 65.8% were female and 34.2% were male. In contrast, 55.7% of those with good physical function were female and 44.3% were male, reflecting a significant gender difference (χ^2^ = 90.706, *p* < 0.001). Furthermore, participants with poor physical function were more likely to have lower levels of educational attainment. Specifically, 62.6% had education levels below primary school, 19.6% had completed primary school, 12.7% had completed middle school, and only 5.1% had attained high school education or higher. In comparison, among those with good physical function, 43.1% had education levels below primary school, 24.4% had completed primary school, 21.7% had completed middle school, and 10.8% had attained high school education or higher (χ^2^ = 362.881, *p* < 0.001).

**Table 1:** Baseline characteristics of participants according to physical function status.

Variables, *n* (%)	Physical function (*N* = 9,497)		χ^2^/*t*-statistic	*p*-value
	Poor (*n* = 3,387)	Well (*n* = 6,110)		
Age^a^ (mean ± SD, years)	65.84 ± 9.937	61.51 ± 9.302	481.869	<0.001***
Gender				
Female	2,227 (65.8)	3,405 (55.7)	90.706	<0.001***
Male	1,160 (34.2)	2,705 (44.3)		
Residence				
Rural	2,402 (70.9)	3,730 (61.0)	92.802	<0.001***
Urban	985 (29.1)	2,380 (39.0)		
Education level				
Below Primary School	2,119 (62.6)	2,634 (43.1)	362.881	<0.001***
Primary School	665 (19.6)	1,488 (24.4)		
Middle School	429 (12.7)	1,325 (21.7)		
High School and Above	174 (5.1)	663 (10.8)		
Marital status				
Divorced/Separated/Widowed/Never Married	722 (21.3)	760 (12.4)	130.418	<0.001***
Married/Cohabitated	2,665 (78.7)	5,350 (87.6)		
Economic capacity				
No	3,014 (89.0)	4,654 (76.3)	230.211	<0.001***
Yes	373 (11.0)	1,456 (23.8)		
Sleep duration (h)^b^				
(0, 6]	2080 (61.4)	3,406 (55.7)	81.633	<0.001***
(6, 8]	922 (27.2)	2,190 (35.8)		
>8	385 (11.4)	514 (8.4)		
Drinking				
No	2,566 (75.8)	4,110 (67.8)	67.246	<0.001***
Yes	821 (24.2)	1970 (32.2)		
Smoking				
No	2,693 (79.5)	4,554 (74.5)	29.848	<0.001***
Yes	694 (20.5)	1,556 (25.5)		
Disability				
No	1,472 (43.5)	4,137 (67.7)	529.903	<0.001***
Yes	1915 (56.5)	1973 (32.3)		
Chronic disease				
No	282 (8.3)	1,047 (17.1)	140.522	<0.001***
Yes	3,105 (91.7)	5,063 (82.9)		
Social trust				
No	2,862 (84.5)	5,045 (82.6)	5.823	0.016*
Yes	525 (15.5)	1,065 (17.4)		
Social support				
No	302 (8.9)	703 (11.5)	15.439	<0.001***
Yes	3,085 (91.9)	5,407 (88.5)		
Social participation				
No	2,807 (82.9)	4,629 (75.8)	64.913	<0.001***
Yes	580 (17.1)	1,481 (24.2)		
Reciprocity				
No	2,965 (87.5)	5,125 (83.9)	23.151	<0.001***
Yes	422 (12.5)	985 (16.1)		

^*^p < 0.05, ^**^p < 0.01, ^***^p < 0.001. ^a^The real age is the interview year minus the respondent’s birth year. ^b^This sleep duration represents the average number of hours participants slept each night over the past month.

The baseline data pertaining to the four dimensions of social capital indicate that 84.5% of participants exhibiting poor physical function reported low levels of social trust, in contrast to 82.6% of those demonstrating good physical function (χ^2^ = 5.823, *p* = 0.016). Furthermore, 91.9% of participants with poor physical function received social support, compared to 88.5% of those with good physical function (χ^2^ = 15.439, *p* < 0.001). Additionally, 82.9% of participants with poor physical function did not engage in social participation, whereas 75.8% of those with good physical function reported the same (χ^2^ = 64.913, *p* < 0.001). Moreover, 87.5% of participants with poor physical function did not engage in reciprocity, compared to 83.9% of those with good physical function (χ^2^ = 23.151, *p* < 0.001). These baseline characteristics underscore significant differences between participants with poor and good physical function across various demographic, social capital, and health-related factors.

### 3.2 Association between the social capital, education, and physical function

To examine the relationship between social capital, educational level, and physical function, we conducted a series of Cox proportional hazards regression analyses. The results are presented in Table 2.

**Table 2:** The association between social capital, education, and physical function.

Social capital	Crude model		Model 1		Model 2	
	HR	95%CI	HR	95%CI	HR	95%CI
Social trust						
No	Ref	-	Ref	-	Ref	-
Yes	1.096	(0.999,1.203)	1.090	(0.991,1.198)	1.070	(0.973,1.176)
Social support						
No	Ref	-	Ref	-	Ref	-
Yes	1.209^**^	(1.074,1.361)	0.910	(0.805,1.030)	0.912	(0.806,1.032)
Social participation						
No	Ref	-	Ref	-	Ref	-
Yes	0.745^***^	(0.682,0.815)	0.826^***^	(0.755,0.905)	0.856^***^	(0.675,0.809)
Reciprocity						
No	Ref	-	Ref	-	Ref	-
Yes	0.519^***^	(0.466,0.578)	0.668^***^	(0.597,0.748)	0.700^***^	(0.626,0.784)
Education level						
Below Primary School	Ref	-	Ref	-	Ref	-
Primary School	0.693^***^	(0.635,0.756)	0.805^***^	(0.753,0.881)	0.841^***^	(0.769,0.921)
Middle School	0.549^***^	(0.495,0.609)	0.715^***^	(0.640,0.798)	0.745^***^	(0.667,0.832)
High School and Above	0.466^***^	(0.400,0.544)	0.611^***^	(0.520,0.718)	0.661^***^	(0.563,0.777)

Crude model: No adjustments. Model 1: Partially adjusted model, adjusting for age, sex, residence, and marital status. Model 2: Fully adjusted model, additionally adjusting for smoking, drinking, sleep duration, disability, and chronic disease. HR, hazard ratio; CI, confidence interval. *p < 0.05, **p < 0.01, ***p < 0.001.

Initially, we investigated the relationship between social capital and physical function. The crude model revealed that social trust was not significantly associated with physical function (*HR* = 1.096, *95%CI*: 0.999–1.203). After adjusting for age, sex, residence, and marital status (Model 1), the association remained non-significant (*aHR* = 1.090, *95%CI*: 0.991–1.198). Further adjustments for smoking, drinking, sleep duration, disability, and chronic disease (Model 2) did not alter the results (*aHR* = 1.070, *95%CI*: 0.973–1.176). In the crude model, social support was significantly associated with better physical function (*HR* = 1.209, *95 CI*: 1.074–1.361). However, this association was not significant after adjusting for demographic variables in Model 1 (*aHR* = 0.910, *95%CI*: 0.805–1.030) and remained non-significant in the fully adjusted model (*aHR* = 0.912, *95%CI*: 0.806–1.032). Social participation was significantly associated with better physical function in all models. The crude model showed a strong association (*HR* = 0.745, *95%CI*: 0.682–0.815), which remained significant after adjusting for demographic variables (Model 1: *aHR* = 0.826, *95%CI:* 0.755–0.905) and in the fully adjusted model (*aHR* = 0.856, *95%CI*: 0.675–0.809). Reciprocity was also significantly associated with better physical function. The crude model indicated a strong association (*HR* = 0.519, *95%CI*: 0.466–0.578), which persisted after adjusting for demographic variables (Model 1: *aHR* = 0.668, *95%CI*: 0.597–0.748) and in the fully adjusted model (*aHR* = 0.700, *95%CI*: 0.626–0.784).

In the study examining the relationship between education and physical function, our findings indicate that participants with a primary school education demonstrated significantly better physical function compared to those with an education level below primary school across all models (Crude model: *HR* = 0.693, *95%CI:* 0.635–0.756; Model 1: *aHR* = 0.805, *95%CI*: 0.753–0.881; Model 2: *aHR* = 0.841, *95%CI:* 0.769–0.921). For participants with a middle school education, the results showed a significant association with improved physical function in all models (Crude model: *HR* = 0.549, *95%CI*: 0.495–0.609; Model 1: *aHR* = 0.715, *95%CI*: 0.640–0.798; Model 2: *aHR* = 0.745, *95%CI*: 0.667–0.832). Participants with a high school education or higher exhibited the most favorable physical function outcomes when compared to those with an education level below primary school (Crude model: *HR* = 0.466, *95%CI*: 0.400–0.544; Model 1: *aHR* = 0.611, *95%CI*: 0.520–0.718; Model 2: *aHR* = 0.661, *95%CI*: 0.563–0.777).

The findings indicate that higher levels of social capital and education are associated with improved physical function among middle-aged and older adults. In particular, social participation and reciprocity demonstrated strong associations with physical function, emphasizing the significance of these dimensions of social capital. In addition, higher educational attainment consistently correlated with enhanced physical function.

### 3.3 Moderating effect of education

The results of the moderating effect of education on the relationship between social capital and physical function are presented in Table 3. The interaction terms for social trust (*aHR* = 1.120, *95%CI*: 1.012–1.240), social participation (*aHR* = 0.923, *95%CI*: 0.840–0.980), and education were significant. However, the main effect of social trust was not significant when compared to social participation. While there is a direct joint effect of education and social trust, it does not suggest a moderating effect of education on the relationship between social trust and physical function.

**Table 3:** Moderating effects of education between social capital and physical function.

Social capital	Model 1		Model 2		Model 3	
	HR	95%CI	HR	95%CI	HR	95%CI
Social trust						
Main effects	1.047	(0.952,1.151)	0.972	(0.867,1.089)	1.071	(0.972,1.181)
Moderator	0.866^***^	(0.831,0.903)	0.789^***^	(0.718,0.867)	0.867^***^	(0.831,0.904)
Interaction term	-	-	1.120^*^	(1.012,1.240)	1.120^*^	(1.012,1.240)
Social support						
Main effects	0.920	(0.813,1.041)	0.896	(0.767,1.048)	0.924	(0.815,1.047)
Moderator	0.866^***^	(0.830,0.902)	0.839^**^	(0.739,0.951)	0.865^***^	(0.830,0.902)
Interaction term			1.036	(0.908,1.182)	1.036	(0.908,1.182)
Social participation						
Main effects	0.880^**^	(0.804,0.964)	0.938	(0.835,1.054)	0.876^**^	(0.800,0.959)
Moderator	0.870^***^	(0.834,0.907)	0.885^***^	(0.845,0.928)	0.870^***^	(0.835,0.907)
Interaction term	-	-	0.923^**^	(0.840,0.980)	0.923^**^	(0.840,0.980)
Reciprocity						
Main effects	0.714^***^	(0.638,0.800)	0.754^***^	(0.651,0.873)	0.716^***^	(0.640,0.801)
Moderator	0.871^***^	(0.836,0.908)	0.879^***^	(0.841,0.919)	0.869^***^	(0.833,0.906)
Interaction term	-	-	0.941	(0.844,1.050)	0.941	(0.844,1.050)

All models are fully adjusted model, additionally adjusting for smoking, drinking, sleep duration, disability, and chronic disease. Reference is no in all four dimensions of social capital. Model 1: Cox regression without interactions. Model 2: Moderated effects Cox model. Model 3: Centered error correction moderated effects Cox model. *p < 0.05, **p < 0.01, ***p < 0.001.

The analysis demonstrates that education plays a significant moderating role in the relationship between specific dimensions of social participation and physical function. Higher levels of education enhance the positive impact of social participation on physical function. However, the benefits of social support and reciprocity on physical function remain consistent across various education levels.

### 3.4 Subgroup analysis

To further investigate the relationship between social capital, education, and physical function, we conducted subgroup analyses based on gender (male vs. female) and place of residence (urban vs. rural). Table 4 provides a visual representation of the overall findings and highlights the differences in the subgroup analyses that are inconsistent with the overall results. In examining the relationship between social capital and physical functioning, we found that female participants’ social support contributed to maintaining good physical function (*aHR* = 0.857, *95%CI*: 0.737–0.998). Additionally, social trust was identified as a risk factor for better physical function in the urban population compared to the overall participants (*aHR* = 1.330, *95%CI*: 1.135–1.560). Social participation was not significantly associated with physical function in the rural population (Supplementary Tables S2–S5). When exploring the moderating effect of education on the relationship between social capital and physical function, we found that the positive moderating effect of education level did not extend to the rural population (Supplementary Tables S6–S9). In addition, we used multiple interpolation methods to address missing data. The overall conclusions, both before and after interpolation, remain consistent, demonstrating the robustness of our findings (Supplementary Table S10).

**Table 4:** Results of the analysis based on subgroups (gender and type of residence).

	Total	Male	Female	Urban	Rural
Cox model					
Social trust	N	N	N	−	N
Social support	N	N	+	N	N
Social participation	+	+	+	+	N
Reciprocity	+	+	+	+	+
Moderating effect					
Social trust	N	N	N	N	N
Social support	N	N	N	N	N
Social participation	P	P	P	P	N
Reciprocity	N	N	N	N	N

Interpretation of the results is based on the Cox fully adjusted model and centered error correction moderated effects Cox model. The + sign indicates that the factor is a protective factor for physical functioning status. The - sign indicates that the factor is a risk factor for the functional status of the body. *p* indicates that the level of education is a promoter. The remaining statistically insignificant results are indicated by N.

## 4 Discussion

This study indicates that social participation and reciprocity promote better physical function among middle-aged and older adults in China and that educational attainment has a moderating effect on the relationship between social capital and physical function.

Social participation and reciprocity are important protective factors for physical function, with positive effects may arising from two key mechanisms. Firstly, increased social interaction plays a crucial role. Active participation allows middle-aged and older adults to connect, share experiences, and provide emotional support, reducing loneliness and enhancing psychological satisfaction. As previous research has shown, strong social relationships are linked to better mental health, effectively lowering depression and anxiety (25, 26). Moreover, increased opportunities for physical activity are vital to the positive effects of both. Social activities often involve physical exercise, such as exercise classes, community gatherings, or group activities. These not only promote physical exercise but also enhance the physical function and daily living abilities of middle-aged and older adults (27, 28). In addition, reciprocity in social relationships fosters a sense of belonging and mutual support. When individuals feel that they can both give and receive help, it enhances their emotional well-being and reduces stress. Previous research indicates that providing social support to others enhances the well-being of older adults more than receiving support (29, 30). Social participation serves as a protective factor for physical functioning in urban areas but shows no significant effect in rural areas. This difference may arise from urban environments providing more engagement opportunities and diverse social networks that encourage participation. In contrast, rural areas may have fewer resources and established norms that restrict social interactions and participation (31, 32).

Social trust and social support show little correlation with the physical function of middle-aged and older adults in China. However, further analysis of urban–rural differences revealed that social trust is a risk factor for the physical function of urban residents, while it has no impact on rural residents. A possible reason is that as individuals migrate from rural areas to cities, their social networks and support systems change significantly (33). In rural areas, residents typically inhabit close-knit communities with high social trust and frequent interactions. In contrast, urban migration poses several challenges. First, urban environments are often marked by isolated interpersonal relationships and reduced interactions, which can decline social trust, as newcomers may not be familiar with their neighborhoods (34). Second, urban life is fast-paced and increasingly competitive. Many immigrants may overlook social interactions while pursuing economic opportunities, intensifying loneliness and isolation, which can negatively impact both mental health and physical function (33, 35).

Social support is a protective factor for women’s functional status, which may be closely tied to their caregiving roles in families and society. Women often juggle multiple roles, such as mother, wife, and caregiver, which increases their responsibilities and pressure (36). Financial support from family or social networks aids in coping with life challenges, significantly reducing anxiety and depression, thereby improving physical function and quality of life. In contrast, men are often seen as economic providers, relying less on external support and more on personal resources to manage stress (37, 38). Consequently, the positive effects of financial support are less pronounced for men.

As the hypothesized, educational attainment moderates the relationship between social capital and physical function among middle-aged and older adults in China, primarily influencing the dimension of social participation. This may be because individuals with higher educational attainment are more likely to recognize the value of social engagement and actively seek opportunities to participate in community activities (39, 40). Furthermore, education equips individuals with the skills and confidence to engage in social networking, reinforcing the benefits of social participation on physical health outcomes (41). Further investigation into these mechanisms could enhance our understanding of how educational attainment shapes social behaviors and impacts well-being.

This study has several limitations. First, the absence of a universally accepted definition of social capital complicates accurate measurement. Additionally, the reliance on existing data from CHARLS to construct measurement variables for social capital may not fully encapsulate its complexity. Self-reported measures of social participation and reciprocity are also susceptible to social desirability bias, which can affect the accuracy of the findings. Furthermore, the dependence on self-reported data regarding disease and life history in the CHARLS survey may introduce recall bias or misclassification errors, complicating the interpretation of results. Finally, the predominantly Chinese sample may restrict the generalizability of the findings. Future research should employ diverse measurement methods for social capital and include a wider range of cross-cultural samples to investigate these relationships more comprehensively and to validate the generalizability of the results.

Given the significant impact of social participation and reciprocity on the physical health of middle-aged and older adults in China, along with the moderating effect of education, it is recommended that community-based initiatives be developed to enhance social engagement and educational opportunities. These initiatives could include the establishment of senior activity centers that provide a variety of social and physical activities, as well as the promotion of lifelong learning programs, such as the University for the Older Adult, aimed at improving social networking skills (42). Additionally, customized interventions that consider gender and residential differences should be implemented. To address the lack of facilities in rural areas, mobile services could be introduced, including bookmobiles, mobile health clinics, and mobile education programs (43, 44). These services can regularly visit rural communities to provide residents with the resources and support they need. Furthermore, fostering inter-sectoral collaboration among health, education, and social service departments could further enhance the health and well-being of this demographic. By implementing these strategies, we can effectively leverage social capital and education to improve physical functioning and overall quality of life for middle-aged and older adults.

## 5 Conclusion

This study investigated the associations between social capital, education, and physical function among middle-aged and older adults in China, utilizing data from the China Health and Retirement Longitudinal Study (CHARLS). Our findings highlight the significant roles of social participation and reciprocity in promoting better physical function. Education plays a significant moderating role in the relationship between specific dimensions of social participation and physical function. The results of this study have important implications for public health policies and interventions aimed at enhancing the physical function of middle-aged and older adults. Promoting social participation and reciprocity, along with improving educational opportunities, can significantly contribute to better health outcomes in this population. Tailored interventions that consider gender and residential differences are essential to address the unique needs of various subgroups.

## Data Availability

The datasets presented in this study can be found in online repositories. The names of the repository/repositories and accession number(s) can be found at: the data from the 2018 and 2020 China Health and Retirement Longitudinal Study (CHARLS) are publicly available at: https://charls.charlsdata.com/pages/data/111/en.html, accessed on 16 November 2023.

## References

[1] United Nations. World population ageing 2019 (ST/ESA/SER.A/444). New York: Department of Economic and Social Affairs, population division (2020).

[2] United Nations. World population prospects 2019: Highlights (ST/ESA/SER.A/423). New York: Department of Economic and Social Affairs, population division (2019).

[3] Berkman LF Glass T Brissette I Seeman TE. From social integration to health: Durkheim in the new millennium. Soc Sci Med. (2000) 51:843–57. doi: 10.1016/S0277-9536(00)00065-4, PMID: 10972429

[4] Cao J Rammohan A. Social capital and healthy ageing in Indonesia. BMC Public Health. (2016) 16:631. doi: 10.1186/s12889-016-3257-9, PMID: 27449022 PMC4957842

[5] Kim Y Schneider T Faß E Lochbaum M. Personal social capital and self-rated health among middle-aged and older adults: a cross-sectional study exploring the roles of leisure-time physical activity and socioeconomic status. BMC Public Health. (2021) 21:48. doi: 10.1186/s12889-020-10043-6, PMID: 33407300 PMC7789776

[6] Norstrand JA Xu Q. Social capital and health outcomes among older adults in China: the urban-rural dimension. Gerontologist. (2012) 52:325–34. doi: 10.1093/geront/gnr072, PMID: 21746837

[7] Ramlagan S Peltzer K Phaswana-Mafuya N. Social capital and health among older adults in South Africa. BMC Geriatr. (2013) 13:100. doi: 10.1186/1471-2318-13-100, PMID: 24073666 PMC3851859

[8] Shen Y Yeatts DE Cai T Yang PQ Cready CM. Social capital and self-rated health among middle-aged and older adults in China: a multilevel analysis. Res Aging. (2014) 36:497–521. doi: 10.1177/0164027513505624, PMID: 25651318

[9] Coll-Planas L Del Valle GG Bonilla P Masat T Puig T Monteserin R. Promoting social capital to alleviate loneliness and improve health among older people in Spain. Health Soc Care Community. (2017) 25:145–57. doi: 10.1111/hsc.12284, PMID: 26427604

[10] Kim Y Radoias V. Social capital and health in developing countries: the case of Indonesia. Soc Indic Res. (2024) 174:1007–24. doi: 10.1007/s11205-024-03422-8

[11] Wind TR Villalonga-Olives E. Social capital interventions in public health: moving towards why social capital matters for health. BMJ Publishing. (2019) 73:793–5. doi: 10.1136/jech-2018-211576 31142609

[12] Uphoff EP Pickett KE Cabieses B Small N Wright J. A systematic review of the relationships between social capital and socioeconomic inequalities in health: a contribution to understanding the psychosocial pathway of health inequalities. Int J Equity Health. (2013) 12:54–12. doi: 10.1186/1475-9276-12-54 23870068 PMC3726325

[13] Xin Y Ren X. Social capital as a mediator through the effect of education on depression and obesity among the elderly in China. Int J Environ Res Public Health. (2020) 17:3977. doi: 10.3390/ijerph17113977, PMID: 32512694 PMC7312359

[14] Jiang J Song J. Health consequences of online social capital among middle-aged and older adults in China. Appl Res Qual Life. (2022) 17:2277–97. doi: 10.1007/s11482-021-10033-9, PMID: 35035602 PMC8741545

[15] Zhao Y Hu Y Smith JP Strauss J Yang G. Cohort profile: the China health and retirement longitudinal study (CHARLS). Int J Epidemiol. (2014) 43:61–8. doi: 10.1093/ije/dys203, PMID: 23243115 PMC3937970

[16] Wang P Chen X Gong J Jacques-Tiura AJ. Reliability and validity of the personal social capital scale 16 and personal social capital scale 8: two short instruments for survey studies. Soc Indic Res. (2014) 119:1133–48. doi: 10.1007/s11205-013-0540-3

[17] Xu H Zhang C Huang Y. Social trust, social capital, and subjective well-being of rural residents: Micro-empirical evidence based on the Chinese general social survey (CGSS). Human. Soc. Sci. Commun. (2023) 10:1–13. doi: 10.1057/s41599-023-01532-1

[18] Chen B. The acquaintance society and reciprocity-based behavior pattern In: The underworld of rural China: Research on rural gangs on China’s Jianghan plain and Dongting Lake. Plain: Springer (2023). 25–49.

[19] Mohd TAMT Yunus RM Hairi F Hairi NN Choo WY. Social support and depression among community dwelling older adults in Asia: a systematic review. BMJ Open. (2019) 9:e026667. doi: 10.1136/bmjopen-2018-026667, PMID: 31320348 PMC6661578

[20] Levasseur M Lussier-Therrien M Biron ML Raymond É Castonguay J Naud D. Scoping study of definitions of social participation: update and co-construction of an interdisciplinary consensual definition. Age Ageing. (2022) 51:afab215. doi: 10.1093/ageing/afab215 35134843 PMC9383398

[21] Klenk C Albrecht J Nagel S. Social participation of people with disabilities in organized community sport: a systematic review. Ger J Exerc Sport Res. (2019) 49:365–80. doi: 10.1007/s12662-019-00584-3

[22] Terry R Townley G. Exploring the role of social support in promoting community integration: an integrated literature review. Am J Community Psychol. (2019) 64:509–27. doi: 10.1002/ajcp.12336, PMID: 31116874

[23] Wilson C Dadswell A Munn-Giddings C Bungay H. The role of participatory arts in developing reciprocal relationships amongst older people: a conceptual review of the literature. J. Aging Soc. Change. (2019) 9:1–16. doi: 10.18848/2576-5310/CGP/v09i04/1-16

[24] Aalen OO Røysland K Gran JM Ledergerber B. Causality, mediation and time: a dynamic viewpoint. J R Stat Soc Ser A Stat Soc. (2012) 175:831–61. doi: 10.1111/j.1467-985X.2011.01030.x, PMID: 23193356 PMC3500875

[25] Kawachi I Berkman LF. Social ties and mental health. J Urban Health. (2001) 78:458–67. doi: 10.1093/jurban/78.3.458, PMID: 11564849 PMC3455910

[26] Węziak-Białowolska D Białowolski P Lee MT Chen Y VanderWeele TJ McNeely E. Prospective associations between social connectedness and mental health. Evidence from a longitudinal survey and health insurance claims data. Int J Public Health. (2022) 67:67. doi: 10.3389/ijph.2022.1604710 PMC9218058 35755953

[27] Millard J. The health of older adults in community activities. Working With Older People. (2017) 21:90–9. doi: 10.1108/WWOP-09-2016-0024

[28] Bertera EM. Physical activity and social network contacts in community dwelling older adults. Act Adapt Aging. (2003) 27:113–27. doi: 10.1300/J016v27n03_08

[29] Thomas PA. Is it better to give or to receive? Social support and the well-being of older adults. J Gerontol B Psychol Sci Soc Sci. (2010) 65B:351–7. doi: 10.1093/geronb/gbp113 20028952

[30] Liang J Krause N Bennett JM. Social exchange and well-being: is giving better than receiving? Psychol Aging. (2001) 16:511–23. doi: 10.1037/0882-7974.16.3.511, PMID: 11554527

[31] Vogelsang EM. Older adult social participation and its relationship with health: rural-urban differences. Health Place. (2016) 42:111–9. doi: 10.1016/j.healthplace.2016.09.010, PMID: 27755999 PMC5116414

[32] Wang R Chen Z Zhou Y Shen L Zhang Z Wu X. Melancholy or mahjong? Diversity, frequency, type, and rural-urban divide of social participation and depression in middle- and old-aged Chinese: a fixed-effects analysis. Soc Sci Med. (2019) 238:112518. doi: 10.1016/j.socscimed.2019.112518 31473574

[33] Meng X Xue S. Social networks and mental health outcomes: Chinese rural–urban migrant experience. J Popul Econ. (2019) 33:155–95. doi: 10.1007/s00148-019-00748-3

[34] Franck KA. Friends and strangers: the social experience of living in urban and non-urban settings. J Soc Issues. (1980) 36:52–71. doi: 10.1111/j.1540-4560.1980.tb02035.x

[35] Skeldon R. Rural-to-urban migration and its implications for poverty alleviation. Asia-Pac Popul J. (1997) 12:3–16. doi: 10.18356/cd2c964e-en, PMID: 12292421

[36] Barnett RC Baruch GK. Women's involvement in multiple roles and psychological distress. J Pers Soc Psychol. (1985) 49:135–45. doi: 10.1037/0022-3514.49.1.135 4020611

[37] Xie Y. (2013). Gender and family in contemporary China.

[38] Zhan HJ. Through gendered Lens: explaining Chinese Caregivers' task performance and care reward. J Women Aging. (2004) 16:123–42. doi: 10.1300/J074v16n01_09, PMID: 15149928

[39] Gong HJ Hong JE. Does postsecondary education attainment matter in community service engagement? Evidence from across 18 OECD countries. Educ Sci. (2021) 11:96. doi: 10.3390/educsci11030096

[40] Ferrer-Esteban G Mediavilla M. The more educated, the more engaged? An Analysis of Social Capital and Education. IRPN. (2017). doi: 10.2139/ssrn.3091171

[41] Yen IH Moss NE. Unbundling education: a critical discussion of what education confers and how it lowers risk for disease and death. Ann N Y Acad Sci. (1999) 896:350–1. doi: 10.1111/j.1749-6632.1999.tb08138.x 10681919

[42] Chen X Giles J Yao Y Yip W Meng Q Berkman L. The path to healthy ageing in China: a Peking University–lancet commission. Lancet. (2022) 400:1967–2006. doi: 10.1016/S0140-6736(22)01546-X, PMID: 36423650 PMC9801271

[43] Sikes S. Rural public library outreach services and elder users: a case study of the Washington County (VA) public library. Public Libr Q. (2020) 39:363–88. doi: 10.1080/01616846.2019.1659070

[44] Park H Tseng H Park M. Exploring online health information-seeking behaviours among older adults in rural areas. Technium Soc Sci J. (2021) 24:235.

